# The Value of Screening for Bicuspid Aortic Valve in First Degree Family Members

**DOI:** 10.7759/cureus.13201

**Published:** 2021-02-07

**Authors:** Emad Kandah, Atefeh Kalantary, Nouraldeen Manasrah, Adan Madadha, Rebecca Pratiti

**Affiliations:** 1 Internal Medicine, McLaren Health Care, Michigan State University, Flint, USA; 2 Internal Medicine, Sinai-Grace Hospital - Detroit Medical Center, Detroit, USA; 3 Diagnostic Medical Laboratories, University of Jordan, Cell Therapy Center, Amman, JOR

**Keywords:** bicuspid aortic valve, endocarditis, screening, aortic aneurysm, abscess

## Abstract

A 54-year-old male with a history of hypertension, diabetes, and sleep apnea presented with a two-week history of dyspnea. The patient was hypoxic with bilateral leg edema. Initial workup showed elevated troponin at 0.15 ng/mL, brain natriuretic peptide of 720 pg/mL, and hyponatremia. Chest X-ray revealed lungs infiltrates with possible pneumonia. An electrocardiogram showed sinus tachycardia and ST depression in septal leads. He received diuretics and antibiotics for fluid overload and pneumonia. Blood culture showed methicillin-sensitive staphylococcus aureus (MSSA). Transthoracic echocardiogram (TTE) revealed a left ventricle ejection fraction (LVEF) of 55-60%, a bicuspid aortic valve (BAV) with mild aortic stenosis and calcification, and an ascending aortic aneurysm of 4.2 cm, though no vegetations. A transesophageal echocardiogram (TEE) demonstrated the BAV, 1.4 cm mobile vegetation, an abscess on the aortic annulus, severe aortic regurgitation, and 4.6 cm ascending aortic aneurysm. He underwent aortic valve replacement, ascending aortoplasty, and coronary artery bypass grafting. He was discharged with eight weeks of antibiotics after a good recovery with resolution of fever, dyspnea, and bacteremia. His son was diagnosed with BAV earlier. Consequently, by screening echocardiogram and education, our patient could have avoided complications of severe infective endocarditis.

## Introduction

Bicuspid aortic valve (BAV) is the most common congenital heart defect with a prevalence of 0.5-2% in the general population and a reported incidence of 1-2% of live births [1]. BAV is a risk factor for aortic stenosis, regurgitation, infective endocarditis (IE), thoracic aortic aneurysm (TAA), and dissection with its associated significant morbidity and mortality [2]. BAV shows some genetic components with an autosomal dominant pattern, incomplete penetrance, and variable expressivity [1, 3, 4]. Multiple genes are associated with isolated or syndromic BAV such as NOTCH1, FBN1, TGFBR2, TGFB2. Additionally, aortopathy is reported in 20-30% of family members of patients with BAV [4]. BAV demonstrates genetic heterogeneity by the involvement of mutations in diverse genes encoding transcription factors, extracellular matrix proteins, and signaling pathways that regulate cell proliferation, differentiation, adhesion, or apoptosis [5].

BAV has a wide range of clinical presentations ranging from asymptomatic, incidental findings on imaging to clinically significant complications such as thoracic aortic aneurysm or dissection. A systolic ejection murmur and or click may be present on auscultation. BAV is associated with serious long-term health risks, including progressive aortic valve disease (stenosis or regurgitation) and thoracic aortic aneurysm and dissection in approximately 35 % of individuals, many of whom require life-saving surgical management [4]. BAV-associated aortopathy varies based on BAV morphology. Typical BAV is associated with dilation of the ascending aorta (principally along its convexity) as well as mild to moderate dilation of the aortic root. The atypical BAV is associated with dilation of both the aortic arch and the tubular ascending aorta, with the aortic root being relatively spared. The third BAV morphologic variant is associated with isolated dilation with aortic root [6]. 

We present a patient with a first degree relative diagnosed with BAV, thus at risk for BAV, presenting with cough and dyspnea. His presentation was complex, involving findings of acute heart failure, acute coronary syndrome, possible atypical viral pneumonia, and IE. The patient had a complicated course of hospitalization secondary to IE requiring surgery. Timely diagnosis of BAV with aortopathy is important for early surgical intervention to prevent its complication.

## Case presentation

**Figure 1:**
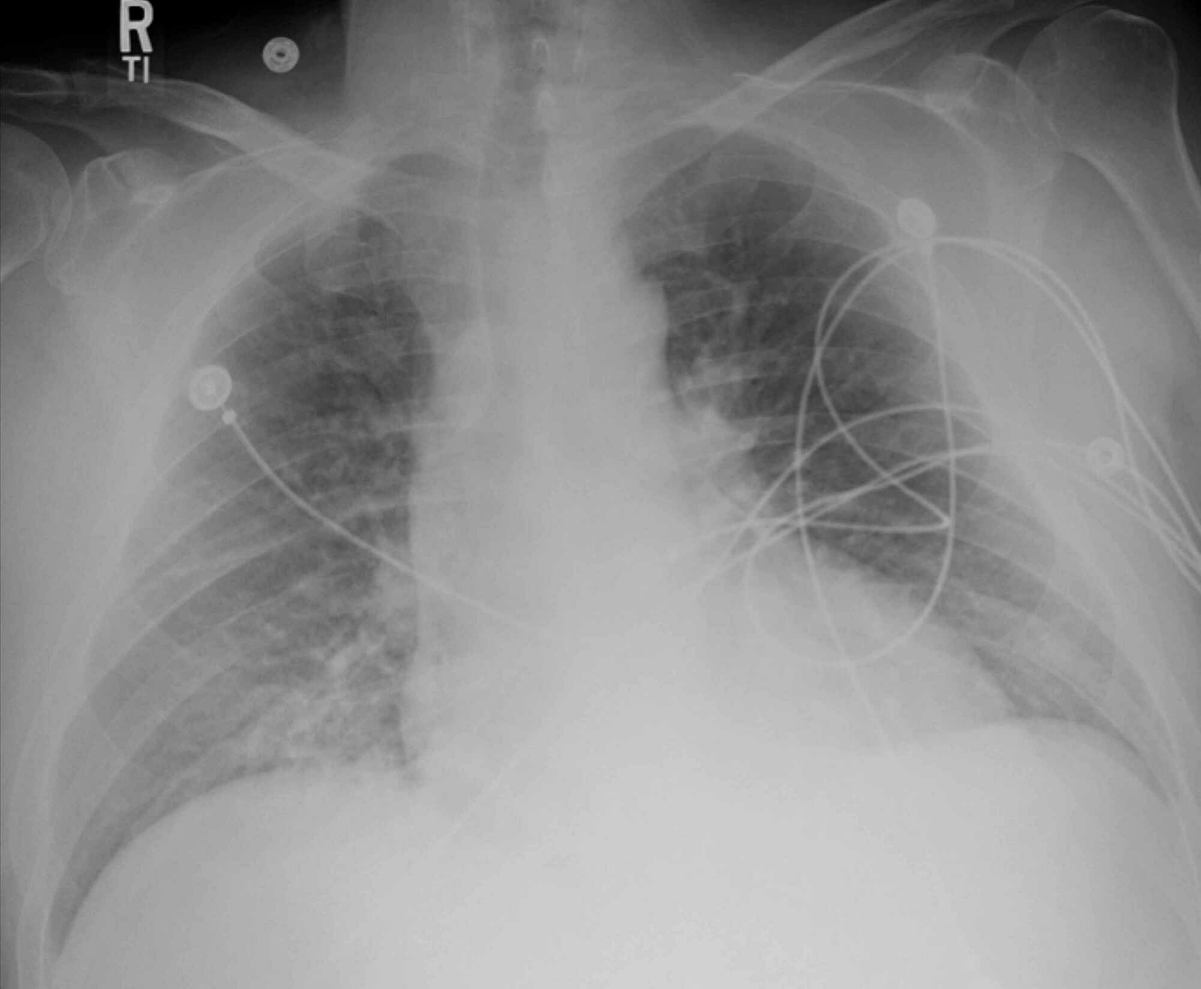
Chest X-ray showing bilateral coarsened interstitial markings and mild lower lung infiltrates

**Figure 2:**
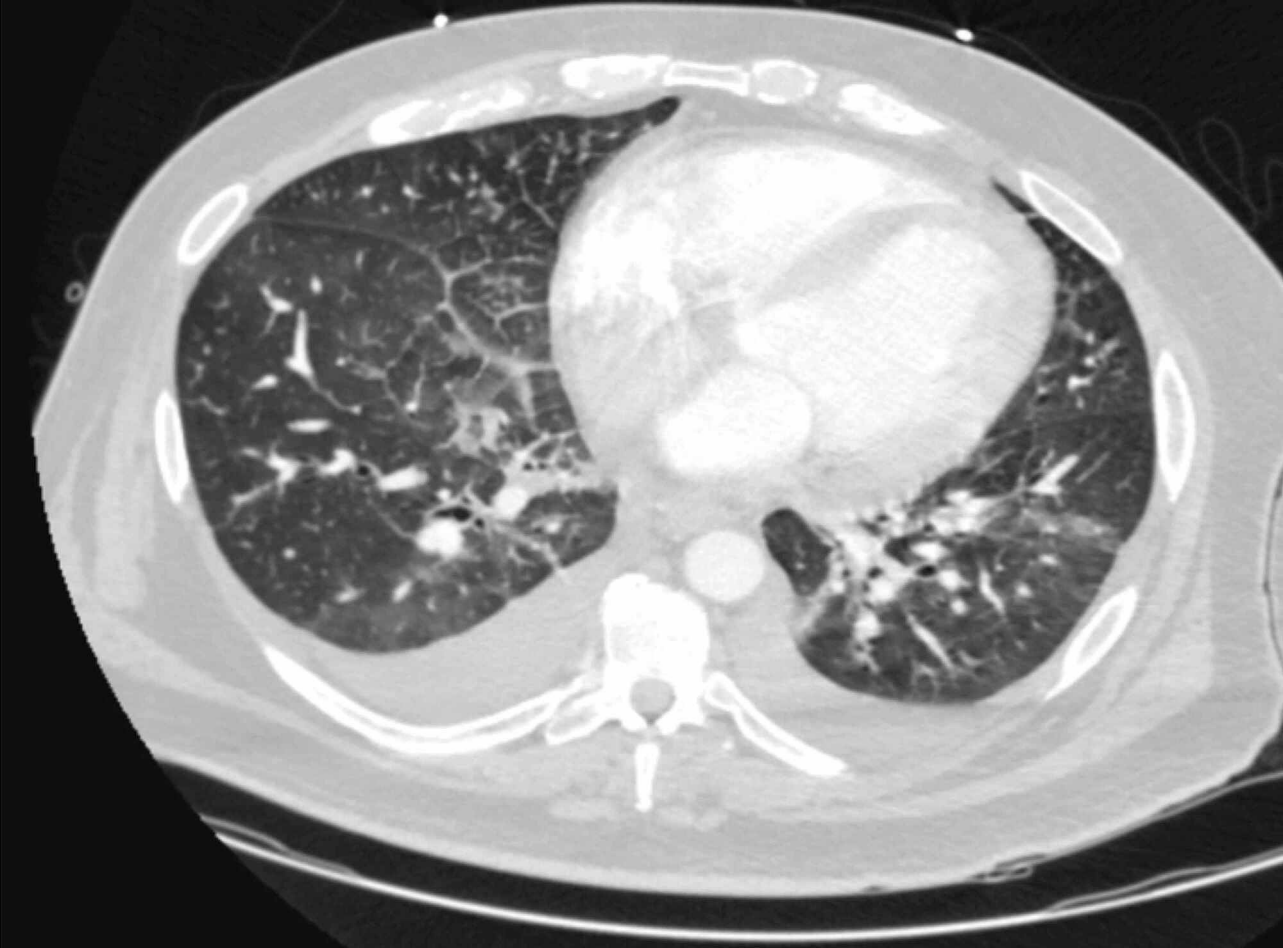
CT scan of the chest with contrast showing ground-glass opacities involving bilateral lower lobes

A 54-year-old male with a past medical history of essential hypertension, type 2 diabetes mellitus, gastroesophageal reflux disease, and obstructive sleep apnea presented to the emergency department with shortness of breath and lower extremity edema for three days. The patient had flu-like symptoms, including cough for two weeks prior to admission. He denied sick contacts or recent travel. History was negative for fever and chills. Other pertinent history revealed an episode of urinary tract infection three weeks prior to presentation. Family history was positive for hypertension, diabetes, and a recent BAV diagnosis in the son. On presentation, patients’ vitals showed a temperature of 102.8 ° F, blood pressure (BP) of 122/59 mm of Hg, heart rate 100/min, respiratory rate 18/min, and oxygen saturation of 98% on room air. Physical examination was positive for mild respiratory distress (respiratory rate of 18 breaths per minute), bilateral basilar crackles, 2+ leg swelling, and a murmur. The patient had a white blood cell count of 15 X 103/µL, sodium 129 mM/L, and a venous lactic acid of 3.4 mM/L. Bilateral coarsened interstitial markings and mild lower lung infiltrate were noted on chest X-ray on admission (Figure 1). CT chest with contrast re-demonstrated the bilateral ground-glass opacities with no evidence of pulmonary embolism (Figure 2).

The patient was initially treated for community-acquired pneumonia (CAP). The patient tested negative twice for severe acute respiratory syndrome coronavirus 2 (SARS-CoV-2) coronavirus disease 2019 (COVID-19) and influenza. The initial troponin level was elevated at 0.15 ng/mL (normal range: 0.00-0,04 ng/ml), with a peak troponin level of 0.20 ng/mL. Additionally, brain natriuretic peptide (BNP) was 720 pg/mL (normal range: 0-100 pg/ml), secondary to fluid overload. Subsequently, the patient was started on diuretics with input and output and blood pressure monitoring.

**Figure 3:**
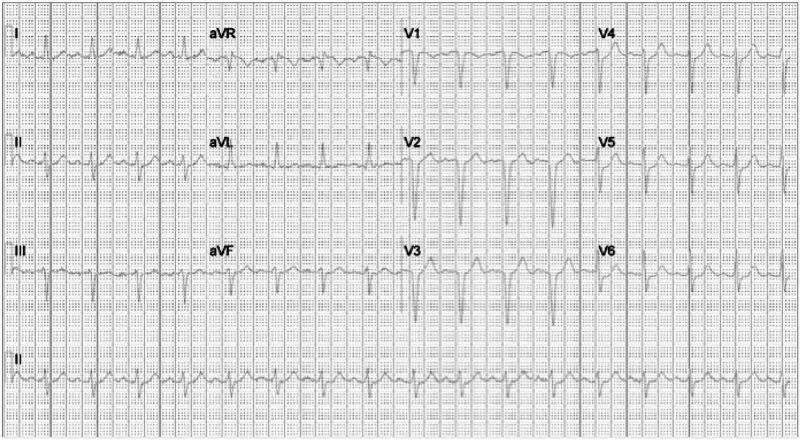
Electrocardiogram showing sinus tachycardia with ST-segment depression in lateral leads V4-V6

**Figure 4:**
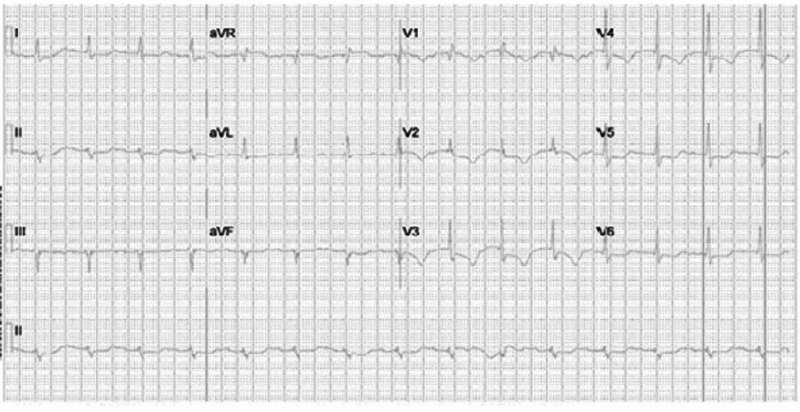
Electrocardiogram showing incomplete right bundle branch block and left anterior fascicular block

Electrocardiogram (EKG) showed sinus tachycardia with ST-segment depression in the lateral lead (Figure 3). Intravenous heparin was initiated. The EKG findings progressed from sinus tachycardia to various conduction abnormalities, including sinus rhythm with first-degree aortic valve (AV) block and incomplete right bundle branch block with a left anterior fascicular block (Figure 4).

**Figure 5:**
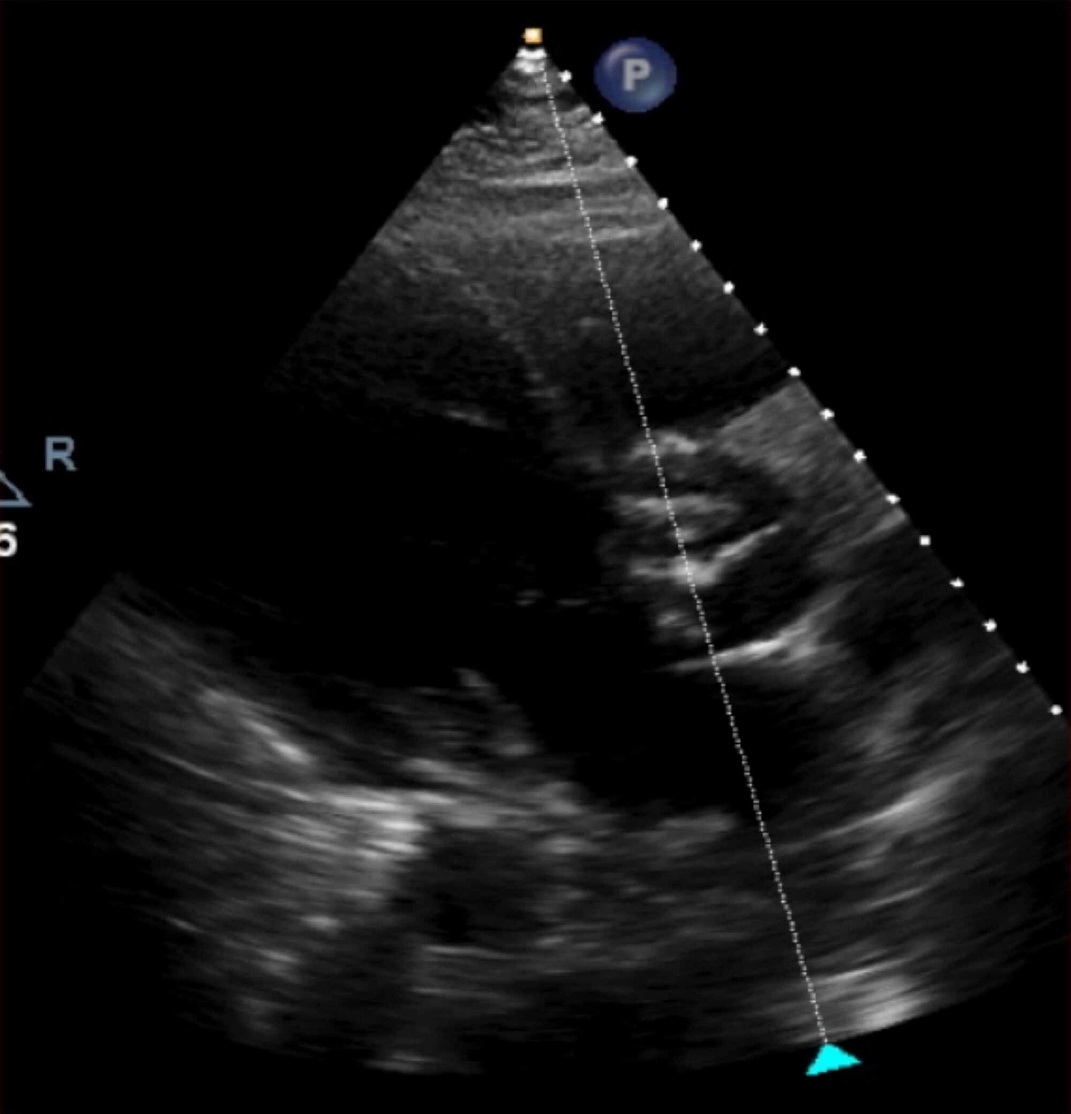
Transthoracic echocardiogram showing bicuspid aortic valve (BAV)

**Figure 6:**
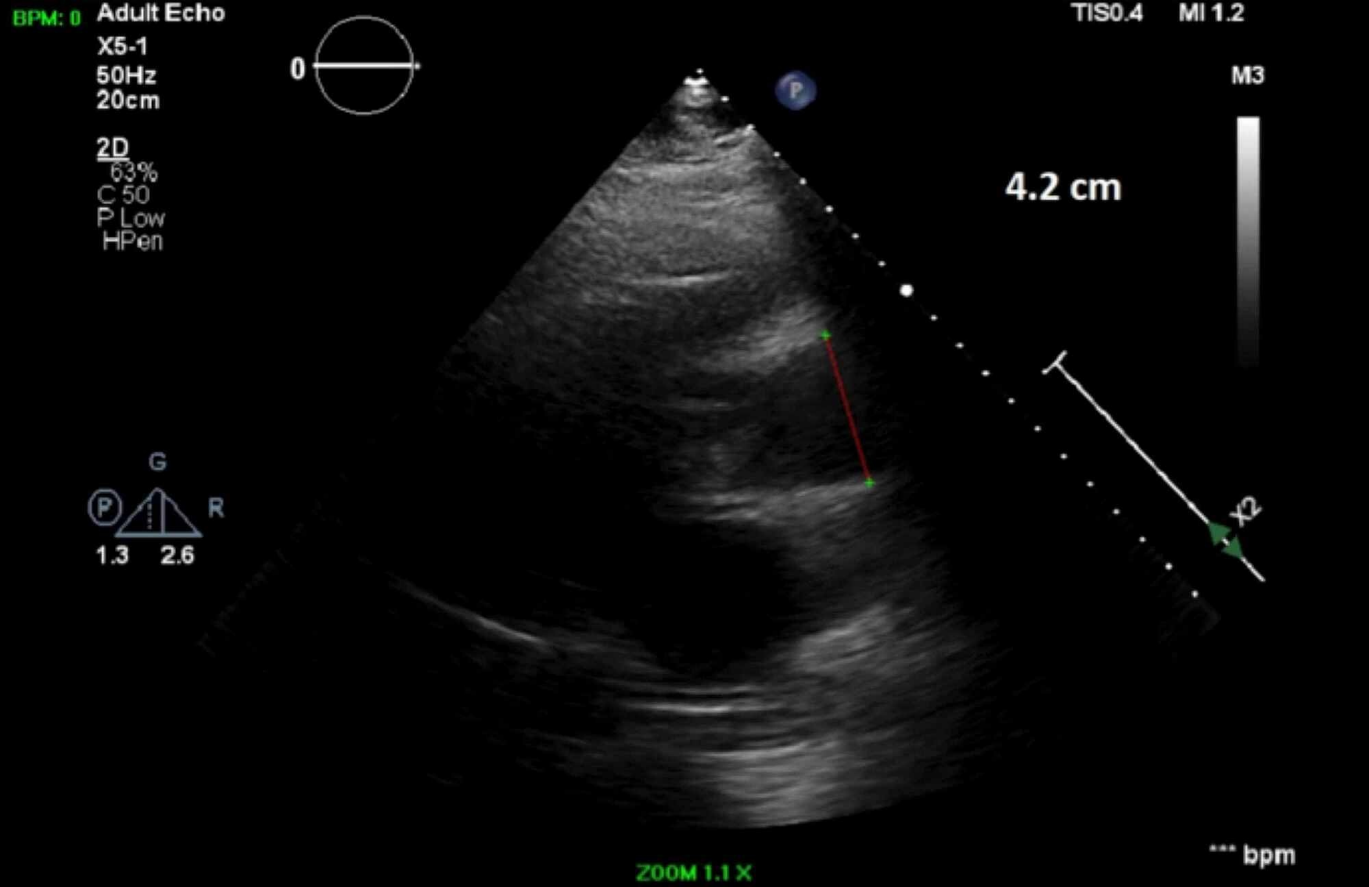
Transthoracic echocardiogram showing dilated ascending aorta of 4.2 cm

**Figure 7:**
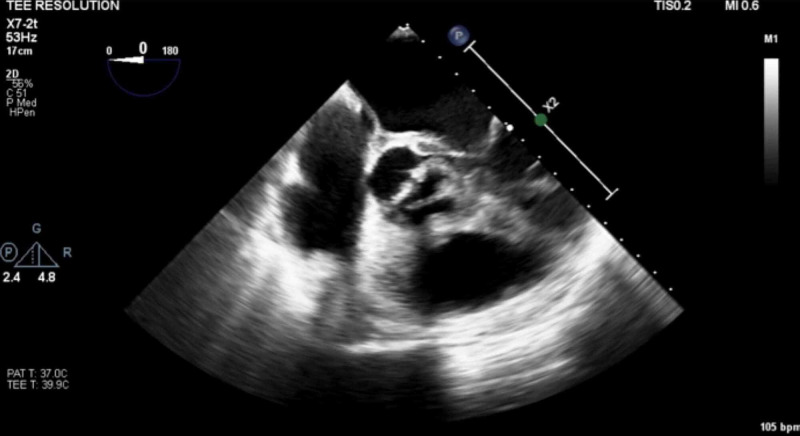
Transesophageal echocardiogram showing bicuspid aortic valve (BAV)

**Figure 8:**
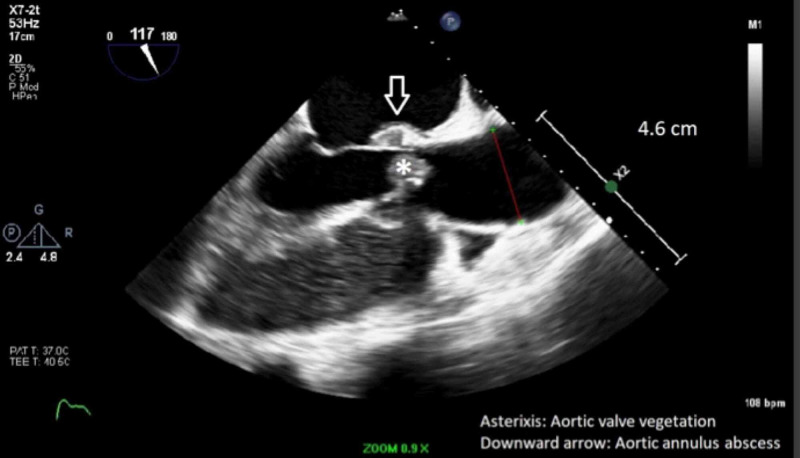
Transesophageal echocardiogram showing AV vegetation (asterisk), aortic annulus abscess (arrow) and dilated ascending aorta of 4.6 cm (red line)

**Figure 9:**
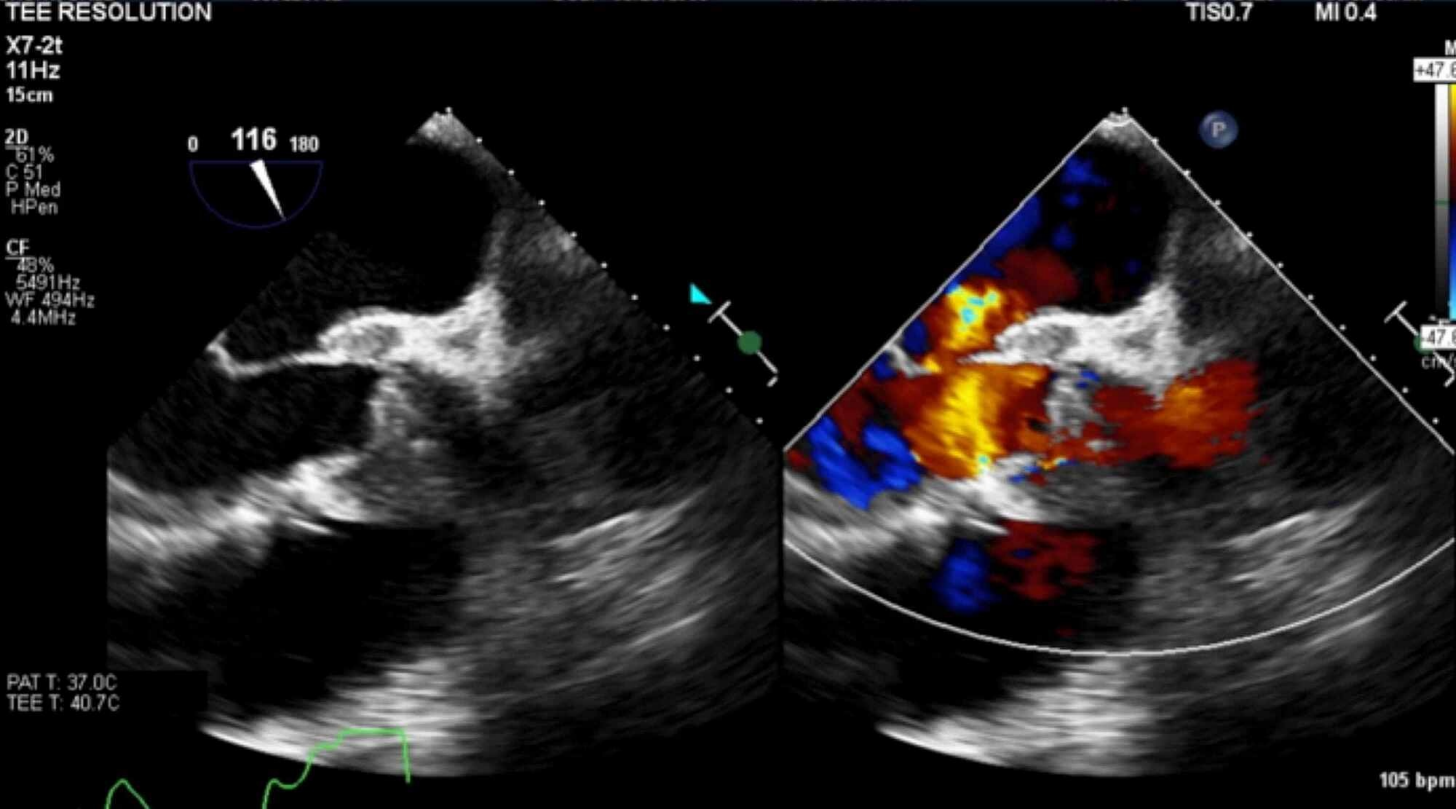
Transesophageal echocardiogram doppler showing severe aortic regurgitation (AR)

Blood cultures grew methicillin-sensitive Staphylococcus aureus (MSSA). TTE showed a left ventricle ejection fraction (LVEF) of 55-60%, a BAV (Figure 5) with mild aortic stenosis and calcification, and an ascending aortic aneurysm of 4.2 cm (Figure 6). Transthoracic echocardiogram (TTE) was indeterminate for vegetations. Patient dyspnea was out of proportion for radiographic findings, so a cardiac etiology was sought. Transesophageal echocardiogram (TEE) demonstrated the BAV (Figure 7), a 1.4 x 1.2 cm posterior leaflet, and 1.5 x 1.4 cm anterior leaflet mobile vegetation of the aorta, aortic annulus abscess, severe aortic regurgitation, and 4.6 cm ascending aortic aneurysm (Figures 8-9). The patient continued to be febrile and bacteremic with worsening hypoxia until surgery. He underwent open-heart surgery with aortic valve replacement (AVR) with a 23 mm bioprosthetic bovine pericardial valve, repair of root abscess with patch closure with a bovine pericardial patch, ascending aortoplasty and coronary artery bypass graft (CABG) with left internal mammary artery to left anterior descending artery. Post-procedure, the patient had a good recovery with resolution of fever and bacteremia. He was discharged home on home health to complete antibiotics with nafcillin for a total of eight weeks.

## Discussion

The bicuspid aortic valve is a structurally abnormal aortic valve that is characterized by the presence of two valve leaflets compared to three leaflets normally present in the aortic valve [1, 2]. BAV has different variants, the most common of which is caused by the fusion of right and left aortic valve leaflets, known as the typical type. This variant represents 70% of all BAV cases. The second most common variant is due to fusion of the right and noncoronary leaflets (the atypical type); it represents 10-20% of BAV cases. A third, less common variant caused by the fusion of the left and noncoronary leaflets is seen in 5-10% of patients with BAV. The true bicuspid aortic valve is a very rare BAV morphology caused by the symmetric fusion of right and left cusps without raphe [6].

BAV-associated aortopathy predominately affects the proximal aorta. Current guidelines recommend a repair of the aortic root/ ascending aorta when the aortic diameter is >55 mm without risk factors and 50 mm in patients with risk factors. Risk factors include root phenotype, predominant aortic regurgitation (AR), uncontrolled hypertension, family history of aortic dissection / sudden death, aortic coarctation, or an aortic growth rate of more than 3 mm per year. American Society of Cardiothoracic Surgery recommends concomitant repair of the root / ascending aorta when the aortic diameter is ≥45 mm in patients undergoing cardiac surgery. Our patient had an aortic root diameter of >45 mm and underwent concomitant aortic root repair with AVR [7]. The surgical repair of the dilated aorta is an important life-saving measure in BAV to prevent life-threatening aortic catastrophe related to acute aortic dissection, which carries a mortality of up to 25%. Incidence of aortic dissection increases 15-fold in the presence of an aortic aneurysm. However, aortic dilatation patterns, the presence of non-modifiable risk factors like aortic growth rate, valve phenotype, family history, and the estimated surgical risk should be considered when planning prophylactic aortic repair [7]. A cooperative study from Japan examined 2,875 patients who underwent thoracic aortic surgery in 36 centers between 2003 and 2005. The research illustrated that in young patients (<65 years of age), outcomes improved with increased hospital volume [3].

Previous case reports have shown the presence of aortic root abscess with conduction disturbances [8]. In one of the reports, aortic root abscess was associated with new-onset complete right bundle branch block (RBBB) with prolonged PR interval, which eventually changed into a left bundle branch block (LBBB) morphology with features of atrioventricular (AV) dissociation followed by alternating bundle branch block on the second day [9]. While in the other case report patient had an aortic root abscess with sinus tachycardia and LBBB in EKG. Our patient had a 1.5 X 1.4 cm abscess in the aortic root requiring surgery. The EKG findings progressed from sinus tachycardia to conduction abnormality of sinus rhythm with first degree AV block and left anterior fascicular block. Eventually patient developed an incomplete right bundle branch block with a left anterior fascicular block. This conduction abnormality could have been related to an aortic abscess. In a multicentric study, as compared to a tricuspid valve, BAV developed more perivalvular abscess requiring early surgery. However, BAV was not associated with higher mortality. Thus, physician counseling about good dental and skin hygiene to BAV patients is important [10].

The risk of endocarditis with BAV is 11 times higher than the general population. This higher risk is related to the inherent native valve abnormality and from prosthetic AVs. Endocarditis is not the most common presenting complication of BAV. In a study including 647 patients with BAV, IE occurred in 13 patients over a follow-up of nine years [11]. Our patient also had a peri annular abscess. Kahveci et al. reported peri annular complications in 64% of BAV IE patients [12]. Additionally, BAV is an independent risk factor for abscess formation in aortic valve infective endocarditis (IE), increasing the likelihood of surgical intervention [9]. According to the American College of Cardiology/American Heart Association Task Force Practice Guidelines, early surgical intervention is indicated in IE in the presence of valve dysfunction causing heart failure, IE caused by resistant organisms including S. aureus, presence of heart block or abscess, or a presence of persistent infection. Our patient met all the criteria and hence underwent urgent surgery for IE [1].

Familial clustering studies have shown BAV is heritable, with a prevalence of 9% in first-degree relatives of patients with BAV and up to 24% in families with >1 person affected [5]. Thus, various ominous outcomes such as bacterial infective endocarditis (IE) and aortic dissection with the high prevalence of BAV in humans signifies the importance of screening for BAV [10]. Hence, the current guideline by the American Heart Association (AHA) recommends screening of first-degree relatives of patients with bicuspid aortic valve with aortic root disease. Fifteen percent of first-degree relatives of BAV patients have BAV on screening, with 1-2% requiring immediate surgery for TAA. Most have cardiac intervention by the fifth decade. Aortic valve stenosis is a more common indication for surgery than aortic insufficiency. Also, a thoracic aortic aneurysm of more than 4.5 cm occurs in 25-45% of patients [2, 3]. Initial assessment of patients with suspected BAV is performed by transthoracic echocardiography. Evaluation should include the aortic valve as well as the ascending thoracic aortic diameter and root measurements. Following the first echocardiographic assessment, interval imaging should be performed in patients with identified BAV to monitor for progressive valvular dysfunction. Surveillance is performed annually if the mean transvalvular gradient is ≥30 mmHg or ascending aortic diameter is ≥40mm, and every other year if the mean gradient is <30 mmHg [2, 3].

## Conclusions

Screening of the first-degree relatives of BAV is often missed. Infective endocarditis could be an uncommon etiology for acute heart failure, acute coronary syndrome, and conductive abnormalities. IE is a less known presentation of BAV. Urgent surgery for IE is indicated in valve dysfunction causing heart failure, IE caused by resistant organisms including S. aureus, presence of heart block, abscess, or persistent infection.
